# Molecular Simulations Reveal the Role of Antibody Fine Specificity and Viral Maturation State on Antibody-Dependent Enhancement of Infection in Dengue Virus

**DOI:** 10.3389/fcimb.2019.00200

**Published:** 2019-06-06

**Authors:** Daniel R. Ripoll, Anders Wallqvist, Sidhartha Chaudhury

**Affiliations:** ^1^Henry M. Jackson Foundation for the Advancement of Military Medicine, Inc. (HJF), Rockville, MD, United States; ^2^Biotechnology HPC Software Applications Institute, Telemedicine and Advanced Technology Research Center, U.S. Army Medical Research and Materiel Command, Frederick, MD, United States

**Keywords:** antibody-virus interactions, dengue virus, antibody dependent enhancement, antibody neutralization, molecular simulations

## Abstract

Recent clinical studies have revealed that severe symptoms of dengue fever are associated with low pre-existing antibody levels. These findings provide direct clinical evidence for the theory of antibody-dependent enhancement of infection (ADE), which postulates that sub-neutralizing levels of antibodies facilitate the invasion of host cells by the dengue virus. Here, we carried out molecular simulations guided by previous *in vitro* experiments and structural studies to explore the role of antibody fine-specificity, viral conformation, and maturation state—key aspects of dengue virology that are difficult to manipulate experimentally—on ADE in the context of primary and secondary infections. Our simulation results reproduced *in vitro* studies of ADE, providing a molecular basis for how sub-neutralizing antibody concentrations can enhance infection. We found that antibody fine specificity, or the relative antibody response to different epitopes on the surface of the dengue virus, plays a major role in determining the degree of ADE observed at low antibody concentrations. Specifically, we found that the higher the relative antibody response to certain cross-reactive epitopes, such as the fusion loop or prM, the greater was the range of antibody concentrations where ADE occurred, providing a basis for why low antibody concentrations are associated with severe dengue disease in secondary infections. Furthermore, we found that partially mature viral states, in particular, are associated with the greatest degree of ADE.

## Introduction

Dengue virus (DENV), a major human pathogen transmitted by *Aedes aegypti* mosquitoes, causes an estimated 390 million infections each year (Bhatt et al., 2013). Four DENV serotypes (DENV1–DENV4), which are found across tropical and sub-tropical regions, vary in prevalence depending on the time and region. Whereas primary dengue infection is typically asymptomatic or results in a mild, uncomplicated fever, secondary infection with a heterotypic serotype is associated with severe disease manifestations, such as dengue hemorrhagic fever, and occasionally, death (Halstead, 1970; Sangkawibha et al., 1984; Guzman and Harris, 2015). This pattern of outcomes has led to the hypothesis that pre-existing immunity to DENV is responsible for enhanced secondary infections.

Recently, two clinical studies that assessed the longitudinal risk of severe dengue disease following primary and secondary infection found that low pre-existing serum concentrations of antibodies (Abs) to dengue virus were associated with the highest risk of severe symptoms. In a study of 3,451 children in Thailand, Salje et al. (2018) found that individuals developed a stable set-point titer within 1 year of a primary infection, and that individuals with pre-existing titers of <1:40 developed hemorrhagic fever at 7.4 times the rate of naïve individuals, compared with 0.0 times for those with titers >1:40. Likewise, in a study of children in Nicaragua, Katzelnick et al. (2017) found that individuals with pre-existing DENV Ab titers within a narrow intermediate range had the highest risk of severe symptoms, compared to those with high DENV Ab titers and those that were seronegative for DENV infection.

The exact mechanism by which pre-existing immunity leads to severe dengue symptoms is unknown. However, *in vitro* studies of dengue infection suggest that Ab-dependent enhancement of infection (ADE) plays a role. In ADE, sub-neutralizing Ab concentrations facilitate viral invasion of host cells via an Fcγ-receptor (FcγR)-mediated mechanism. Specifically, Abs bound to the virus surface engage Fc receptors, resulting in FcγR-mediated endocytosis. Subsequent acidification of the phagocytic vesicles triggers viral membrane fusion and invasion of the host cell. Although *in vitro* studies using both monoclonal and polyclonal Abs have shown that ADE occurs under various conditions for a range of FcγR-bearing cells, major questions remain regarding its physiological role in dengue disease severity.

First, lower set-point titers are associated with severe dengue disease during secondary infection, but not primary infection, suggesting that serotype specificity, in addition to antibody concentration, plays a role in ADE. Second, it is unclear how the same infecting viral titer that is largely asymptomatic in naïve individuals is pathogenic in exposed individuals. In this study, we sought to address these questions by extending a molecular simulation approach to model the roles of antibody concentration, serotype-specificity, and viral heterogeneity in ADE.

DENV infection triggers a broad immune response, which in part involves the production of hundreds to thousands of distinct monoclonal Abs (mAbs) which bind to a range of epitopes on the surface of the virus. Previous *in vitro* studies of flavivirus infection suggest that a stoichiometric threshold of 20–50 Abs bound to the virion is sufficient for neutralization (Pierson et al., 2007). MAbs to DENV are typically classified as type-specific (TS) Abs that bind to and/or neutralize only one serotype, and cross-reactive (CR) Abs that bind to and/or neutralize all four serotypes. An important study by Beltramello et al. (2010) found that activation of immunological memory years after a DENV infection leads to the production of large amounts of broadly CR Abs. However, most of these Abs are incapable of neutralizing infection even at very high concentrations, and only a small quantity of them may exhibit TS or potent neutralizing activity.

A key feature of these poorly neutralizing CR Abs is that they target immunodominant epitopes, such as the fusion loop (FL) of the envelope (E) protein or the *pr* fragment of the prM protein, which have low accessibility or availability. *In vitro* studies have shown that, Abs which bind to these epitopes are highly prone to ADE (Halstead and O'Rourke, 1977; Beltramello et al., 2010; Dejnirattisai et al., 2010; Yeo et al., 2015). Fully mature DENV viral particles do not contain *pr* fragments or present the FL epitope on their surface (Perera and Kuhn, 2008; Zhang et al., 2013). However, infected host cells produce a wide spectrum of viral particles in different maturation states with varying ratios of prM and E, which are manifest in cryo-electron microscope images as spiky patches and heterogeneous morphologies. Recent studies of *in vitro* models of DENV infection have shown that, mAbs which target these epitopes are typically highly cross-reactive, poorly neutralizing, and highly prone to ADE, even when they are produced by infections due to other flaviviruses, such as the Zika virus (ZIKV) (Barba-Spaeth et al., 2016; Stettler et al., 2016).

We previously presented a method (Ripoll et al., 2016) for estimating the stoichiometry of Ab-flavivirus complex formation and modeling antibody-dependent neutralization of dengue virus, using a molecular simulation approach based on the theory of multiple equilibria in proteins (Tanford and Kirkwood, 1957; Beroza et al., 1991). We used a coarse-grained structural representation of the Ab-flavivirus complex based on high-resolution cryo-EM and X-ray crystallography structures that allowed us to capture important structural characteristics, such as the spatial distribution of the epitope around the virion. Here we extended this approach to consider (1) mixtures of CR and TS mAbs that recognize distinct epitopes, and (2) heterogeneous viral populations that include partially mature states in various structural configurations.

## Materials and Methods

We modeled the interaction of a mixture of CR and TS Abs with the envelope of flaviviruses, using an extension of the structure-based Monte Carlo (MC) approach described previously (Ripoll et al., 2016). Here, we highlight the changes we introduced to the procedure to model the binding of an Ab mixture to a virion. First, we assumed that the total Ab concentration, [*Ab*_*tot*_], is given by Equation (1), where [*Ab*_*TS*_] and [*Ab*_*CR*_] are the partial concentrations of TS and CR Abs, respectively.

(1) $$\begin{array}{r} \left[{Ab}_{tot}\right]=\left[{Ab}_{TS}\right]+\left[{Ab}_{CR}\right] \end{array}$$

We assumed that binding occurs as a Brownian-like process where Abs randomly collide with a virus envelope. The virion concentration, [*V*], was considered infinitely dilute (virion-virion interactions are negligible, i.e., [*Ab*_*tot*_] >> [*V*]). Each set of CR or TS Abs was represented by a single mAb with the highest affinity of the respective group, and the chosen representative Abs corresponded to well-studied mAbs whose epitopes were mapped to E or prM, preferably through Cryo-EM or X-ray crystallography experiments.

### Coarse-Grained Structural Model of DENV-Ab Complexes

We used coarse-grained representations of both the Abs and the viral envelope to capture the relevant geometrical features of the complexes. As described above, we generated three-dimensional models of the viral particles by combining homology modeling techniques with existing cryo-EM and X-ray structure data. For the four DENV serotypes, we constructed all-atom models for whole virus envelopes in their immature “spiky,” immature “smooth,” and mature conformations (Zhang et al., 2003, 2013; Perera and Kuhn, 2008; Yu et al., 2008; Kostyuchenko et al., 2013). Using the fully mature or immature models, we produced partially mature viral particles with varying ratios of immature to mature content, 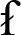, and then converted these into coarse-grained models.

We represented the partially mature state as a sphere of radius *r* [= (*r*_*Imm*_ + *r*_*Mat*_)/2], where *r*_*Imm*_ and *r*_*Mat*_ correspond to the radii of the immature and mature viral envelopes, respectively, derived from all-atom homology models of the viral envelopes in the particular maturation state (i.e., mature, immature *spiky*, and immature *smooth*) (Zhang et al., 2003, 2013; Perera and Kuhn, 2008; Yu et al., 2008; Kostyuchenko et al., 2013). We used a tessellation procedure to partition the surface of the sphere into elements of equal size (Tegmark, 1996), each of which determined a pixel on the sphere, and whose total number determined the resolution of the spherical grid. We derived a simplified representation of an epitope, ξ, on the tessellated sphere from the collection of surface elements intersected by the radial projections of the actual epitope atoms in the three-dimensional model of the partially mature envelope.

At the start of each MC run, the fraction of prM content, *f*, was randomly selected based on a normal distribution centered around 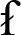, an input parameter defining the mean prM content, and a composite viral particle consisting of E-prM and E subunits (in a ratio corresponding to *f*). The distribution of E-prM and E subunits was either arranged randomly across the whole viral surface (for the “smooth” conformation) or as a contiguous surface patches (for the “rough” conformation). The types of epitope distributions were consistent with available experimental data indicating the existence of partially mature flavivirus particles containing varying amounts of uncleaved prM (Junjhon et al., 2008; Nelson et al., 2008; Dowd and Pierson, 2011), and cryo-EM data showing viral particles where a portion of the surface remains in the immature “spiky” state (Perera and Kuhn, 2008; Junjhon et al., 2010). Finally, CR and TS epitopes were mapped to the corresponding E-prM and E subunits on the viral surface.

Abs were represented as circular “soft disks” that could interact with other Abs through steric interactions and bind to their epitopes. This simplified “footprint” representation removes the need to account for changes in the Ab orientation relative to the virus surface. An Ab was considered *bound* when it landed on a given surface element and occluded a center of one of its epitopes. The radius of the soft disk, *r*_*Fab*_, reflects the overall excluded volume of the Ab. We previously found that a *r*_*Fab*_ of 27.8 Å was sufficient to reproduce the Ab binding stoichiometry for most Ab-flavivirus complexes with available cryo-EM structures (Ripoll et al., 2016).

### Ab-Virus and Ab-Ab Interactions

We used the theory of multiple equilibria in proteins to model Ab binding (Edsall and Wyman, 1958; Steinhardt and Reynolds, 1969; Bisswanger, 2008), where Abs represented the ligands, and the virus envelope represented the macromolecule whose binding sites corresponded to the epitopes of the Abs. We modeled the behavior of a mixture of two types of Abs by adapting a methodology used to study pH titration in proteins (Beroza et al., 1991). During the simulation process, we assumed that Abs stochastically bind to and unbind from binding sites on the virus surface in an epitope-specific manner, through the use of a free energy function that derives the free energy of the virus binding state based on its binding configuration and the binding affinity of each mAb in the system (see Supplemental Materials: section A. Modeling Ab-virus and Ab-Ab interactions). Previously, we used this approach to model the binding of mAbs to DENV (Ripoll et al., 2016), here, we extended this approach to simulate the binding of *mixtures* of two mAbs, a CR and TS mAb, to DENV. The Abs in the system, in addition to binding to the virus surface, were also assumed to sterically interact with each other, potentially occluding neighboring binding sites in the described above.

### Simulating Ab Binding and Neutralization

We carried out simulations for combinations of [*Ab*_*TS*_], and [*Ab*_*CR*_], with each partial concentration ranging from 10^−1^ to 10^−14^ M. For each pair of partial concentrations, we carried out 500 binding simulation runs (~10^8^ M steps each), collecting statistics from 25,000 independent configurations of Abs bound to the viral capsid. From the simulation data, we computed the observed number of bound Abs < *N*_*bound*_ > to generate Ab occupancy curves. Similarly, we computed the mean number of bound CR Abs, < *N*_*CR*_ >, and the mean number of bound TS Abs, < *N*_*TS*_ >.

We generated theoretical curves of infectivity, *r*_*infc*_, or neutralization, *r*_*neut*_ [= (1–*r*_*infc*_)], using a structure-based model of neutralization introduced in our previous work (Ripoll et al., 2016). This model represents a variation of the “multiple hit” model (Parren and Burton, 2001), which assumes that docking of multiple Abs to a single virion is required for neutralization. In previous work we showed that this model shows ~50% neutralization for an average < *N*_*bound*_ > of ~30, which is close to the neutralization threshold postulated by the “coating theory” for flaviviruses.

### Simulating ADE

We defined a quantitative model of ADE that assumes that the enhancement, **E**_**ADE**_, produced by a given virion is determined by two variables: the infectivity of the particle, *ȓ*_*infc*_, and the rate of phagocytosis, ȓ_**phg**_.

(2) $$\begin{array}{r} E_{ADE}={ȓ}_{infc}\cdot {ȓ}_{phg} \end{array}$$

We assumed the rate of phagocytosis to be a function of the number of successful encounters of the virion with the receptors on the surface of the invaded cell. Additionally, we assumed all cells to have the same number of receptors. A successful encounter required the presentation of an Ab by the virus to the host cell, hence, the greater the < *N*_*bound*_ > on the virion, the more likely the encounter was successful. With these considerations, we assumed that ȓ*r*_*phg*_ = *C N*_*bound*_, where *N*_*bound*_ is the number of Abs bound to the particular configuration of the virus, and *C* is a constant.

### Monte Carlo Simulations

To produce a stoichiometric curve for a given dual Ab mixture and virus complex, we need to determine the average < *N*_*bound*_ > at different concentration values of the Ab mixture. To this end, we performed importance sampling using MC methods (Beroza et al., 1991) to simulate the Ab-virus binding process. After first selecting a pair of partial concentration values for the free Abs, [*Ab*_*TS*_] and [*Ab*_*CR*_], we carried out 500 independent simulation runs. Each run started with the initialization of the system, in which the number of CR epitopes for an Ab-free viral envelope was randomly defined using a normal distribution with a mean corresponding to the immature content 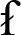 (specified by an input value). During the course of the MC simulation, surface elements were randomly selected along with a type of action (a “trial” move): binding or release of an Ab. For binding moves, the type of Ab was chosen randomly, and a binding attempt was made only when the surface element was associated with an epitope of the correct type. For release moves, the procedure first checked whether an Ab was bound to the surface element under consideration, and upon confirming a bound Ab, attempted to unbind it. Trial moves were accepted and rejected based on the Metropolis criteria.

During a run, which typically ended after 10^8^ MC steps, we collected statistics every 2 × 10^6^ steps. This sampling frequency was determined based on the correlation time between approximately independent measurements as computed using the methodology of Beroza et al. (1991). To produce averages for a given concentration, we used all samples collected from the 500 independent runs.

### Code Availability

The program code and processing scripts are available upon request.

## Results

### Simulating Ab Binding in Flaviviruses

We carried out molecular simulations of polyclonal Abs binding to DENV, West Nile Virus (WNV), and ZIKV virions to explore the contributions of Ab concentration, epitope fine specificity, and virus maturation state on infectivity and ADE. We represented polyclonal sera as a mixture of representative TS and CR mAbs and assigned binding affinities that reflect homotypic and heterotypic specificities. Representative TS and CR Abs were selected from published data where serotype specificity and epitope information were available. In particular, we focused on mAbs where cryo-EM, X-ray crystallography, or shotgun mutagenesis methods were used for epitope mapping (Table 1).

**Table 1:** DENV-specific mAbs used in simulations.

**mAb**	**Specificity**	**Virus**	**Method**	**PDB ID**	**Epitope**	**References**
2D22	TS	DENV2	Cryo-EM	4UIF, 4UIH, 5A1Z	E dimer	De Alwis et al., 2012; Fibriansah et al., 2015
2H2	CR	DENV1-4	Cryo-EM	3J42	prM	Henchal et al., 1982; Wang et al., 2013
5G22	CR	DENV1-4	Shotgun mutagenesis	–	prM	Smith et al., 2012, 2016
EDE2 A11	CR	DENV1-4, ZIKV	X-ray diffraction	4UTB, 5LCV	EDE2^a^	Dejnirattisai et al., 2015; Rouvinski et al., 2015; Barba-Spaeth et al., 2016
E16	TS	WNV	X-ray diffraction	3IYW, 1ZTX	E–DIII	Nybakken et al., 2005; Kaufmann et al., 2010

a *EDE2 stands for “E dimer-dependent epitope” with sensitivity to disruptions in N-linked glycosylation sites at positions 153 and 155 of the DENV E protein*.

**Figure 1:**
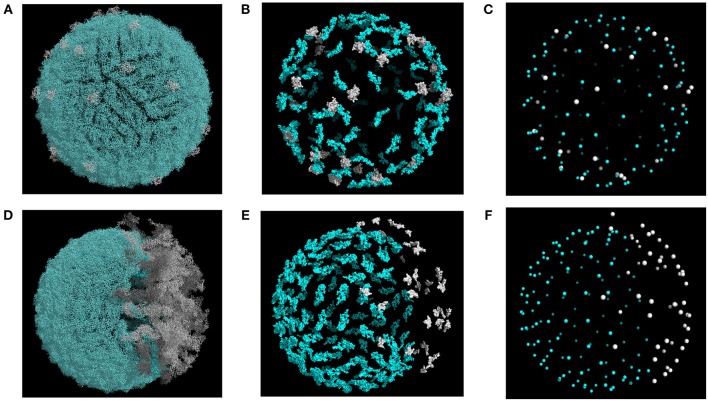
Structural models of DENV envelope used for simulations. Representative structures are shown for smooth **(A–C)** and rough **(D–F)** conformations. Epitopes and epitope centers for TS (blue) and CR (white) mAbs are highlighted in **(B,C,E,F)**. Structures were generated with maturation corresponding to a prM content of 20%.

We carried out MC-based simulations using coarse-grained representations of the flavivirus envelope and mAbs. Specifically, we started from a high-resolution structural model of the virion, mapped the appropriate mAb epitopes onto the virion surface, and then reduced the virion representation to that of a tessellated sphere, with the epitope residues defined as points. Each Ab was modeled as soft disk that represents its binding “footprint,” and Abs could interact with one another through steric interactions (Ripoll et al., 2016). We modeled viral structural heterogeneity by varying the degree of maturation and by modeling “smooth” (Figures 1A–C) and “rough” (Figures 1D–F) conformational states. A given virion in the model assumed a range of maturation states, each defined by the fraction of prM-E heterodimers present in the model, from fully immature (100%) to fully mature (0%).

During simulations, each run started from a coarse-grained model of the partially mature virion. Representative models are shown in Figures 1C,F for the smooth and rough partially mature states, respectively. In the smooth form of the virion, prM-E heterodimers were distributed randomly over the virus envelope. We used the smooth form of the WNV envelope, together with WNV mAb E16, to explore the effects of epitope exposure. The smooth form of a DENV virion was also used in simulations that assumed simultaneous binding of CR and TS Abs. In the latter case, we chose DENV mAb 2D22, which binds to E protein dimers (Fibriansah et al., 2015), as a representative of TS mAb, and DENV mAb 5G22, which binds to an epitope on prM, as a representative CR mAb.

For the rough form of the virion, we considered mosaic structures from cryo EM experiments which show partially mature particles containing E-prM heterodimers in the immature spiky state (Junjhon et al., 2010). These heterodimers aggregate over the virus surface to form a single immature patch (Figure 1D). For the rough virion, we used the mAb 2H2, which binds a pr epitope, as a representative CR mAb, along with 2D22 as a representative TS mAb. Figures 1B,E show the atomic representations of TS and CR epitopes in the rough form of the virus. Simulations of the rough form were also used to investigate the effect of partial maturation on the binding of a CR mAb of the EDE2 family, namely mAb 747(4) A11, which targets E dimer epitopes in DENV and ZIKV (Dejnirattisai et al., 2015, 2016; Barba-Spaeth et al., 2016).

### Ab Binding Stoichiometry in Partially Mature Virions

We first carried out simulations in which we varied the concentration of a single type of mAb that binds to partially mature virions either in the smooth or rough state, to observe the impact of reduced epitope exposure on infection, neutralization, and ADE. We considered a wide range of maturation states (prM content from 0 to 100%) and ran the simulations with TS mAb concentrations ranging from 10^−1^ to 10^−14^ M, assuming high binding affinity for the virion (K_A_ = 10^−9^ M). At each mAb concentration, we carried out 500 independent simulations, each 10^8^ steps long, and evaluated the mean number of bound Abs. From the simulation trajectories, we computed the average infectivity and average enhancement.

**Figure 2:**
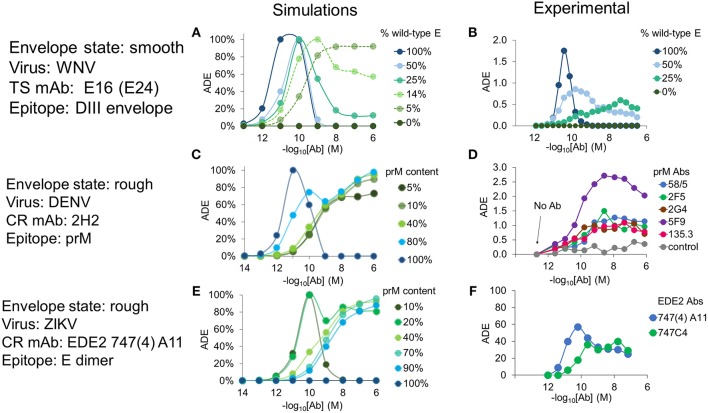
Effects of epitope exposure on Ab activity—experiments vs. simulations. Plots of ADE as a function of Ab concentration for viral particles displaying varying numbers of epitopes. **(A)** Simulated ADE using virions in the smooth state, at various ratios of wild-type to mutated E proteins forming the virus envelope. **(B)** Experimental ADE data for WNV mAb E24, obtained from Pierson et al. (2007). **(C)** ADE predicted by simulations of anti-prM Ab 2H2 binding to epitopes exposed on mosaic particles at different degrees of maturation. **(D)** Enhancement of DENV2 infection of primary monocytes in the presence of human anti-prM antibodies; data from Dejnirattisai et al. (2010). **(E)** ADE predicted by simulations of mAb EDE2 747(4)-A11 binding to mosaic particles of ZIKV at different degrees of maturation. **(F)** Enhancement of infection in human myeloid cell U937 by ZIKV strain HD78788 in the presence of variable concentrations of EDE2 mAbs 747(4)-A11 and 747C4; data from Dejnirattisai et al. (2016). Simulated ADE values were normalized by the maximum ADE observed in that condition.

Pierson et al. (2007) carried out *in vitro* experiments on the effects of epitope exposure in the related flavivirus WNV. In one experiment, they investigated Ab occupancy requirements for virus infectivity by controlling the number of epitopes of a type-specific Ab (mAb E24) displayed by recombinant WNV particles. They achieved this by mixing wild-type WNV E protein, which contains the E24 epitope, with mutant WNV E-prM heterodimers, which include a point mutation that abrogates E24 binding. To simulate this experiment, we used published structural information from mAb E16 whose epitopes closely overlap with those of mAb E24 (Pierson et al., 2007). For simulation purposes, we assumed that these mixed particles were in the smooth state (Figure 1A), containing wild-type E molecules in the mature state and mutant E-prM in the immature state. We found good agreement between our simulated results and the experimental data with respect to ADE and relative infectivity at varying levels of maturation. Increasing epitope exposure (by increasing the percentage of wild-type E in the particles) led to a reduction in the overall infection rate, as found in the experiments (Figures S1A,B). The range of concentrations where ADE occurred broadened and shifted toward higher concentrations as epitope exposure decreased (Figures 2A,B).

DENV glycoproteins organized on the envelope surface co-exist in two forms, mature dimers and immature trimers (Junjhon et al., 2008, 2010; Plevka et al., 2011), which form mosaic “rough” viral particles that are often released by infected host cells. We carried out simulations using this rough conformation (Figure 1B) for a range of maturation states. We first explored the binding of CR Abs to immature DENV using epitope information for the anti-prM mAb 2H2 (Wang et al., 2013) and compared it with the experimental results of Dejnirattisai et al. (2010), which showed that anti-prM Abs are prone to elicit ADE even at very high Ab concentrations (see Figure 2D). Our simulations showed similar results: unlike with an anti-E mAb, such as E24, 2H2 exhibited ADE across a broad range of Ab concentrations and maintained high ADE even at very high concentrations (Figure 2C).

Finally, we examined a recently studied class of CR mAbs that target the EDE2 epitope (Dejnirattisai et al., 2015). EDE2 Abs can strongly neutralize DENV, but some members of the family enhance infection of ZIKV (Dejnirattisai et al., 2016). The epitope of EDE2 Abs is a conformational one that forms when the virus matures and exposes E dimers on the envelope surface. We used structural information for the mAb EDE2 747(4)A11 bound to DENV and ZIKV to define the epitopes (Rouvinski et al., 2015; Barba-Spaeth et al., 2016). Our simulations for EDE2 mAb 747(4)A11 in complex with the rough form of ZIKV showed peak ADE activity for highly mature particles (prM content < 10%) and monotonically increasing ADE at lower Ab concentrations at a wide range of maturation states (prM content >10% and <90%) (Figure 2E). Both of these ADE characteristics are reproduced qualitatively as in experimental observations (Figure 2F).

### Neutralization and ADE in Primary DENV Infections

To explore the role of ADE in secondary heterotypic DENV infections, we used a semi-quantitative approach to describe differences in host immune status between primary and secondary infections. We assumed that changes in the neutralization properties of blood sera between primary and secondary infections are mainly related to variations in the binding affinity of the Abs. In our model, variations in binding affinity are specified through changes in the dissociation constants of Abs, *K*^*TS*^ and *K*^*CR*^. To model blood sera conditions that follow a primary or secondary homotypic infection, we first produced stoichiometric plots for dual mixtures of CR and TS Abs assuming that the affinity of the TS Ab is comparable to that of the CR counterpart (*K*^*TS*^≈*K*^*CR*^). Conditions associated with a secondary heterotypic infection, on the other hand, were modeled assuming a substantial loss in affinity of TS Abs with respect to CR Abs (*K*^*TS*^≫*K*^*CR*^).

**Figure 3:**
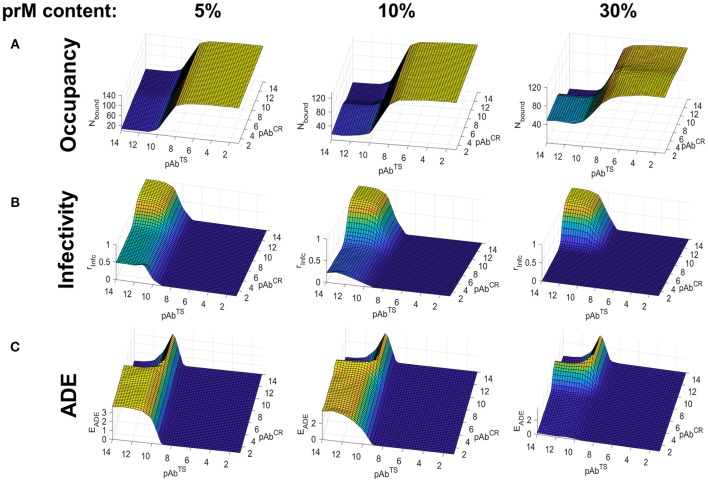
Simulated Ab binding occupancy, neutralization, and ADE in primary DENV infection using the smooth virus conformation. Estimated Ab binding occupancy **(A)**, infectivity **(B)**, and ADE **(C)** at a range of TS and CR Ab concentrations for highly mature (5% prM content), moderately mature (10% prM content), and low-maturity (30% prM content) virions during a primary or homotypic infection.

We carried out binding simulations at a range of TS and CR Ab concentrations (10^−2^ to 10^−14^ M) and immature (prM) contents ranging from 5 to 40%, using the smooth conformation of the virus. Figure 3A shows the Ab occupancy as a function of Ab concentration and maturation state. For highly mature virions (5–10% prM content), CR epitopes, which are found on prM, contributed little to the total occupancy even at high CR Ab concentrations where the epitopes are saturated, and overall Ab occupancy was primarily driven by TS Ab concentration. At lower maturation levels (30 and 40% prM content), CR Abs played a greater role in overall Ab occupancy, although TS Ab concentration continued to be the primary contributing factor. This is because, TS epitopes greatly outnumbered CR epitopes, even at higher maturation states.

Figure 3B shows infectivity as a function of Ab concentration and maturation state. At high maturation states, it was determined almost entirely by the TS Ab concentration, as the number of CR epitopes to drive neutralization was insufficient. At lower maturation states (20–40% prM content), CR Abs and TS Abs contributed comparably to neutralization, and infectivity was only seen in conditions of low CR and TS Ab concentrations.

Figure 3C shows ADE as a function of Ab concentration and virus maturation state. We calculated ADE as a function of both Ab occupancy and Ab neutralization, with the peak ADE at an Ab occupancy just under the neutralization threshold. For highly mature virions (5% prM content), ADE occurred in conditions corresponding to low TS Ab concentration and high CR Ab concentration. Under these Ab concentration conditions, ADE peaked for a prM content of around 10% and became almost negligible for a prM content above 20%. At low maturation states (prM content ≥30%), ADE only occurred in a narrow range of Ab concentrations corresponding to the transition between high occupancy and no occupancy.

Overall, for primary infections, Ab occupancy and neutralization was primarily driven by TS Ab concentration, and ADE occurred only at a low TS Ab concentration and a moderate to high CR Ab concentration in highly mature virions. In virions of low to moderate maturation states, ADE rarely occurred, and was restricted to a very narrow range of Ab concentrations whenever it did.

### ADE in Heterotypic Secondary DENV Infections

We extended our model to capture secondary heterotypic DENV infection. We assumed that TS Abs produced in a primary infection had low binding affinity against a heterotypic virion, while the binding affinity of CR Abs was similar between homotypic and heterotypic infections. To reproduce these conditions, we chose dissociation constants of *K*^*TS*^ = 1*E*^−5^*M* for TS Abs and *K*^*CR*^ = 1*E*^−9^*M* for CR Abs in heterotypic infections.

**Figure 4:**
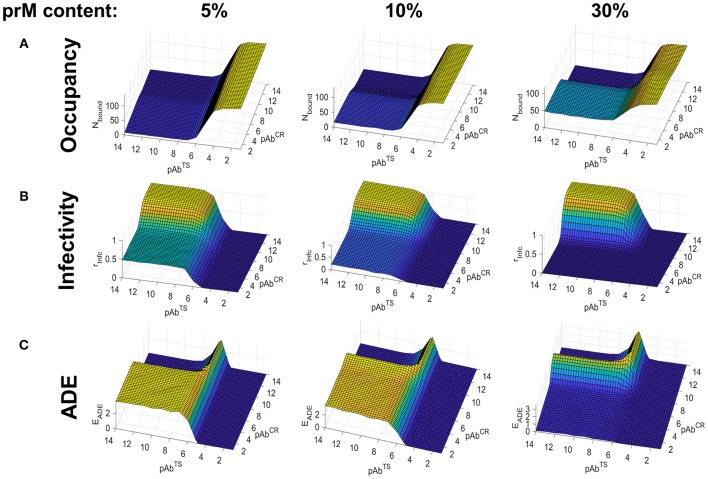
Simulated Ab binding occupancy, neutralization, and ADE in heterotypic secondary DENV infection using the smooth virus conformation. Estimated Ab binding occupancy **(A)**, infectivity **(B)**, and ADE **(C)** at a range of TS and CR Ab concentrations for highly mature (5% prM content), moderately mature (10% prM content), and low-maturity (30% prM content) virions during a heterotypic infection.

We carried out binding simulations under the same conditions as in the case of the primary infection. Figure 4A shows Ab occupancy as a function of the partial concentrations of TS and CR Abs and the maturation state of the virus. For highly mature virions (5% prM content), occupancy was determined primarily by TS Ab concentration because of the low numbers of CR epitopes. Unlike in the primary infection, however, appreciable Ab occupancy occurred only at high TS Ab concentrations, due to the poor binding affinity of TS Abs to a heterotypic serotype. At moderate to low levels of viral maturation (prM content > 10%), CR Ab concentration plays a significant role in determining Ab occupancy, owing to the high binding affinity for its epitope.

Figure 4B shows infectivity as a function of partial TS and CR Ab concentrations and viral maturation state. For highly mature virions (5% prM content), neutralization was only observed at high concentrations of TS Ab, owing to its poor binding affinity for the heterotypic serotype. Under conditions of low TS and high CR concentrations, only virions with a low maturation state (prM content ≥ 30%) exposed enough CR epitopes to become fully neutralized. On the other hand, virions with moderate to high maturation (prM content ≤ 10%) were only partially neutralized.

Figure 4C shows ADE as a function of partial TS and CR Ab concentration and viral maturation state. For the highly mature virion, ADE occurred at a wide range of TS and CR Ab concentrations. This is because the low affinity of TS Abs and the low epitope availability of CR Abs led to sub-neutralizing Ab occupancy at a wide range of concentrations. As in the case of a primary infection, under conditions of low TS and high CR concentrations, ADE peaked at a prM content of around 10% and became negligible at a prM content above 20%. For moderate to highly immature virions, ADE occurred at a narrow range of concentrations, limited to conditions where Ab occupancy was >0 but sub-neutralizing.

Overall, whereas ADE occurred mainly during conditions of very low TS Ab concentration in primary or homotypic infection, it occurred under a wide range of TS Ab concentrations in heterotypic secondary infection.

### Role of Rough Viral Conformation in ADE

To explore how different types of partially mature virus particles affect infectivity and ADE, we next carried out a series of simulations in which a fraction of the viral envelope was in the immature rough or spiky state during a heterotypic infection (see Figure 1D). Unlike previous simulations of the smooth form of the virus where CR Abs targeted pr epitopes randomly distributed on the virion surface, here we used simulations of the rough form of the virus in which CR Abs targeted exposed pr epitopes forming a single patch on the surface of the virion.

**Figure 5:**
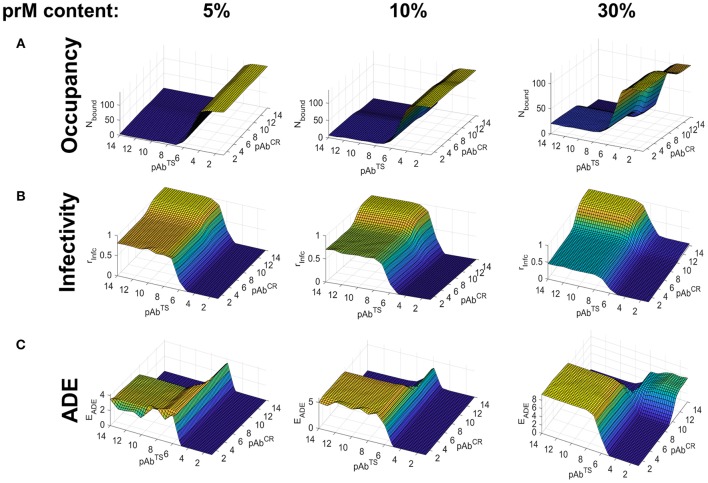
Simulated Ab binding occupancy, neutralization, and ADE in heterotypic secondary DENV infection using the rough virus conformation. Estimated Ab binding occupancy **(A)**, infectivity **(B)**, and ADE **(C)** at a range of TS and CR Ab concentrations for highly mature (5% rough conformation), moderately mature (10% rough conformation), and low-maturity (30% rough conformation) DENV virions during heterotypic infection.

Compared to the simulations of heterotypic infection using the smooth form of the virion, we found several differences. Most notably, occupancy and neutralization were driven almost entirely by the partial concentration of TS Abs, and CR Abs had virtually no neutralization capacity, even at high concentrations (Figures 5A,B). Furthermore, the degree of maturation had little impact on the neutralization capacity of CR Abs within the range of maturation states considered (prM content of 5–40%). In contrast to heterotypic infection in the smooth virion, the neutralization capacity of CR Abs increased as the prM content increased, eventually providing a level of neutralization comparable to TS Abs at high levels of virus immaturity (Figure 5B).

Finally, whereas ADE occurred only in highly mature virions in the case of the smooth virion, it occurred at all levels of viral maturation in the rough virion (Figure 5C). Together, these findings suggest that the rough viral conformation may be particularly prone to ADE under a wide range of conditions during a secondary heterotypic infection.

### Simulating Longitudinal Risk of ADE

Recent long-term pediatric cohort studies (Katzelnick et al., 2017; Salje et al., 2018) based on large groups of individuals have shown that the risk of severe dengue disease is correlated with low anti-DENV antibody titers in the blood, with risk being significantly lower for children having high antibody titers, and, surprisingly, for seronegative individuals. We sought to use our simulation results to explore alternative scenarios and conditions under which an individual might be prone to enhancement of dengue disease. In particular, we investigated how time-dependent changes of Ab concentrations and other variables determine DENV infection and disease enhancement as the outcome.

In a pediatric dengue cohort study, Katzelnick et al. (2017) showed that serum titers over time could vary substantially from one individual to another. In a separate study, Salje et al. (2018) found that the time-dependent behavior of TS and CR Ab concentrations in an individual during primary and post-primary infections could be modeled as a sharp increase in titers followed by an exponential decay. They found that, after the first year, titers tend to stabilize to a set-point titer.

**Figure 6:**
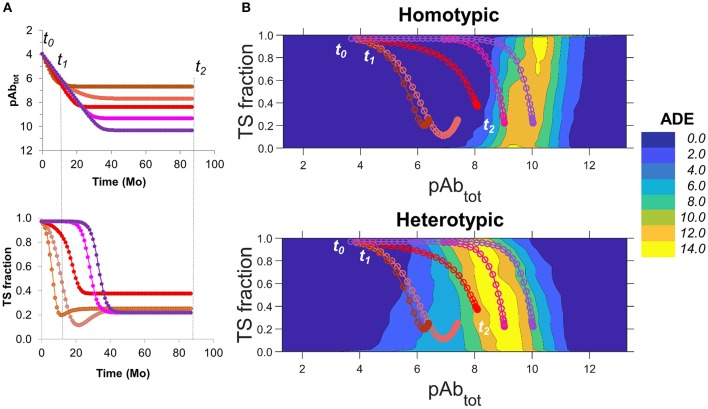
Estimating the longitudinal risk of ADE from simulation data. **(A)** Hypothetical scenarios of changes in DENV-specific serum Ab concentration over time (top) from the convalescent phase of a primary infection (*t*_0_) to a time point where set-point titers are achieved (*t*_2_), where *t*_1_ indicates 1 year post infection. Scenarios with increasingly higher set-point titers are shown from brown to purple. The bottom panel shows the corresponding TS Ab fractions for each scenario. **(B)** Contour plots for ADE as a function of fine specificity, expressed as TS fraction, and total Ab concentration. Scenarios are plotted using corresponding colors in **(A)** for homotypic (top) and heterotypic (bottom) conditions. Plots are derived from simulations of smooth virions with a prM content of 20%.

To our best knowledge, more detailed data on time-dependent changes in the concentration of CR and TS Abs against DENV from individuals are not publicly available. As such, we used information from the above-mentioned studies to produce hypothetical curves of Ab concentrations as functions of time that capture relevant aspects of the observed experimental behavior. Figure 6A shows hypothetical curves of total Ab concentration and TS Ab fraction as a function of time. In these hypothetical curves, total Ab concentrations are highest at the convalescent phase of a primary infection (*t*_0_), followed by an exponential decay leveling off to a range of set-point titers, months to years later (*t*_2_). We modeled the risk of ADE as a function of the fine specificity of serum Ab (the fraction of the total Ab concentration occupied by TS Abs), and the total Ab concentration from the convalescent phase of a primary infection until set-point titers are achieved (*t*_0_ to *t*_2_) (Figure 6B).

For homotypic infections, the risk of ADE was minimal, even at low Ab concentrations, across all ranges of fine specificity (Figure 6B; top) and time points. As such, the risk of ADE during homotypic secondary infection was minimal regardless of the rate of decay of Ab concentration or the endpoint titer, even for individuals with low set-point titers (top panel of Figure 6B, showing projections of different Ab concentration time courses onto ADE risk).

For secondary heterotypic infections, however, there was a substantial risk of ADE under a wide range of Ab concentrations and fine specificities (Figure 6B; bottom). The risk was particularly pronounced under conditions where the fine specificity of the Ab response is biased toward CR Abs over TS Abs (TS fraction < 0.50), and at intermediate concentrations of total Ab. During the first year after infection (from *t*_0_ to *t*_1_), the risk of ADE was low in all scenarios, as the total Ab concentration was higher than the intermediate concentrations associated with ADE. Thereafter, the outcome varied by scenario. Individuals with very low set-point titers (purple in Figure 6A) were most susceptible to ADE within a specific time window (between 12 and 24 months post-infection), as their serum Ab concentration passed through the high-risk intermediate range. By contrast, for individuals with higher set-point titers (magenta and red in Figure 6A), long-term risk of ADE remained high after the first year, as their Ab titers stabilized within the high-risk intermediate range. Finally, individuals with very high set-point titers (pink and brown in Figure 6A) were almost entirely free of risk. Finally, these trends were robust for both smooth and rough virions differing widely in prM content.

## Discussion

Here, building on previous work, we developed a model of Ab binding to the flavivirus surface in order to determine the molecular and structural basis for ADE. We validated this model with experimental *in vitro* data on antibody occupancy and relative infection from a range of studies (Ripoll et al., 2016). We used smooth conformation of the flavivirus virion to model infection and ADE as a function of Ab concentration and epitope accessibility. Our simulations show infection curves similar to those obtained by Pierson et al. (2007) finding that virions with low epitope exposure are able to avoid neutralization entirely, while virions with epitope exposures >30% showed a typical neutralization curve. Our simulations reproduced general features observed in experimental studies of ADE, such as the bell-shaped curve for high degrees of mature content (Pierson et al., 2007) and the displacement of maximum ADE toward high concentrations with diminishing epitope exposure. Our model predicted ADE to occur over a wide range of high Ab concentrations at an epitope accessibility below 30%. At high Ab concentrations, it predicted some degree of ADE at an epitope accessibility below 25%, and predicted maximum ADE to occur around an epitope accessibility of 5–10%.

In addition, we explored the role of partial maturation on infectivity by modeling viral particles in their “rough” state (i.e., mosaic particles). Comparison of our computational results with the limited experimental data on the neutralizing capacity of CR anti-prM and EDE Abs revealed that our simulations captured the relevant features of infectivity and ADE observed in experiments. Our simulations also linked observed patterns of infection, neutralization, and ADE to specific conditions that determine epitope accessibility: Ab affinity, Ab concentration, and mature content of the virus. The simulations predicted that rough and smooth viral particles produce different patterns of infectivity and ADE for similar levels of maturation. For the smooth particle, anti-EDIII Abs were fully neutralizing at high Ab concentrations ([Ab] >> K_D_) and induced ADE only at sub-neutralizing concentrations ([Ab] ~ K_D_). By contrast, for the rough particle, both EDE2 and anti-prM Abs induced ADE even at high Ab concentrations for a wide range of maturation states. These trends were robust to changes in maturation state (prM content of 25–90%).

We modeled the polyclonal Ab response as a combination of CR and TS Abs and modeled heterotypic infection by reducing the binding affinity of the TS Ab while maintaining that of the CR Ab. Neutralization and ADE were significantly affected by conditions associated with heterotypic infection. In homotypic infection, neutralization is driven primarily by TS Abs and ADE is limited to conditions of very low TS Ab concentration. In heterotypic infection, both TS and CR Abs generally contributed to neutralization, but in a suboptimal fashion: CR Abs were limited by epitope accessibility while TS Abs were limited by poor binding affinity. During heterotypic infection involving virions in the smooth conformation, ADE became increasingly pronounced across a wide range of Ab concentrations as the prM content decreased below 20%.

The exact maturation state of DENV *in vivo* is unclear, but *in vitro* studies show that the virus can be produced in a wide range of maturation states. In our simulations, maturation state played a prominent role in neutralization and ADE. At lower levels of maturation (higher prM content), CR Abs specific to pr epitopes increasingly played a role in neutralization. Furthermore, in both homotypic and heterotypic infections, ADE was maximal at a maturation state corresponding to a prM content of 5–10% for smooth virions, and over 30% for rough virions. Previous studies have suggested that unlike some flaviviruses, DENV may have evolved to have suboptimal prM cleavage (Rouvinski et al., 2017). This feature of DENV could contribute to ADE in secondary infections.

We found that the immature rough form of the virus also had interesting characteristics with respect to neutralization and infection. It was poorly neutralized by CR Abs, and unlike the smooth form, where ADE is highest at relatively high levels of maturation (prM content of 5–10%), it was prone to ADE at a wide range of maturation states. In our model, CR Abs are poorly neutralizing in the rough form (relative to the smooth form) because the FL epitopes are tightly clustered in trimeric spikes that prevent full occupancy of all three epitopes for steric reasons. Thus, for a prM content of 40%, the most CR epitopes that a virion can display is 32. Our simulations estimate that the average number of bound CR Abs is generally below the neutralization threshold postulated by the “coating theory” (~30 Abs), even at the highest CR Ab concentrations tested. In the smooth form of the virus, these epitopes are distributed homogenously across the viral surface, with negligible interference between neighboring bound CR Abs. Collectively, our results suggest that the rough form of the virus may be particularly pathogenic in cases of heterotypic infection, where neutralization is driven primarily by CR Abs.

Our finding that the viral maturation state plays a significant role in ADE has implications for *in vitro* and *in vivo* models of ADE. Two recent studies showed discordant results between a *in vivo* and *in vitro* model of ADE where they found that high concentrations of CR mAbs 4G2 and 6B6C-1 show high mortality in the AG129 mouse model, but do not exhibit ADE at high concentrations in an *in vitro* model using THP-1 cells (Watanabe et al., 2015; Ramasamy et al., 2018). They also found that TS mAb 3H5, which shows similar neutralization and ADE characteristics as 4G2 and 6B6C-1 *in vitro*, was highly protective in the AG129 mouse model. One possible explanation for these discrepancies is that the maturation state of virus produced in the *in vivo* model is different from virus produced in the *in vitro* model. In our model, if the prM content of the virion produced *in vitro* is >20%, then a standard bell-shaped ADE curve is to be expected. By contrast, if the prM content of the virion produced in the *in vivo* model is <20%, then ADE would be expected even at high Ab concentrations. Furthermore, this difference in maturation state would be expected to have a greater impact on CR Abs than TS abs, in an epitope-specific manner. Thus, lower immature contents in the virions produced by AG129 mice could explain the high mortality of mice at high Ab concentrations and the discordance between *in vitro* and *in vivo* results. Differences in the conditions inside host cells have been shown to affect the maturation state of newly produced virions (Nelson et al., 2008). Thus, it is conceivable that *in vivo* and *in vitro* conditions alter the virus maturation state, shifting the neutralization and enhancement capacities of CR Abs. Such mechanism could also explain why ADE can overcome the protective efficacy of Abs in a tissue-dependent manner (Watanabe et al., 2015).

### ADE and Severe Dengue Disease

Our ADE model informs a number of recent clinical findings on severe dengue disease in natural infection studies. First, prior studies have shown that low pre-existing antibody levels are associated with an increased likelihood of severe dengue disease only during secondary heterotypic infections (Katzelnick et al., 2017). Our work shows why low pre-existing antibody levels may not enhance secondary *homotypic* infection—namely that highly neutralizing TS Abs generated during primary infection can neutralize a secondary homotypic infection even at low Ab concentrations, precluding the occurrence of ADE.

Second, other studies have shown that after a primary infection, individuals acquire temporary immunity to all four serotypes—an immunity that has been suggested to last anywhere from 6 months to several years (Sabin, 1952; Halstead, 2007; Montoya et al., 2013; Sharp et al., 2014). Our work suggests that this is because serum Ab concentrations are sufficiently high following a primary infection, such that even low affinity (poorly cross-reactive) TS Abs can neutralize a secondary heterotypic infection. As the Ab concentration decays toward a set-point titer, the combination of high-affinity CR Abs that target poorly accessible epitopes, and low-affinity TS Abs that show poor binding occupancy, results in sub-neutralizing binding stoichiometry and a high propensity for ADE. In short, serum conditions conducive to ADE emerge months to years after a primary infection, depending on the subsequent Ab decay rate.

Finally, previous studies have shown that different individuals achieve different set-point titers ~24–36 months after a primary infection (Salje et al., 2018). Our simulations suggest that the risk of ADE for individuals with very low set-point titers temporarily increases as the serum Ab concentration passes through the high-risk range and then falls below that level, whereas the risk for those with higher set-point titers may remain consistently high. This suggests that measuring set-point titers may be sufficient to predict an individual's relative risk of severe dengue disease.

### Implications for Vaccine Research

Recent studies of Dengvaxia, a tetravalent vaccine developed by Sanofi-Pasteur, have revealed that for vaccine recipients with no prior exposure to DENV, there is a modest *increase* in the risk of severe dengue disease (Aguiar et al., 2016). Other studies have shown that poorly immunogenic dengue vaccines, or tetravalent dengue vaccines in which the subject fails to seroconvert in all four serotypes, can result in the induction of CR Abs over TS Abs (Kanesa-Thasan et al., 2001; Gromowski et al., 2018). Our findings suggest that low Ab titers with mostly CR Abs that target only a few epitopes per virion are prime conditions for ADE, supporting the theory that poorly immunogenic dengue vaccines act as a surrogate for post-primary dengue infection. Our results demonstrate that Ab concentration and specificity are critical host determinants of ADE, underscoring the importance of measuring not only antibody titer but also fine specificity when assessing future dengue vaccine candidates. In this respect, our simulations support vaccine designs such as the one recently reported using tetravalent virus-like particles displaying the domain III of E (Ramasamy et al., 2018), in which the antigen contains well-characterized serotype-specific epitopes that are present in large quantities on the virion surface. Finally, our simulations point to the degree of viral maturation as another important determinant of ADE. The fraction of extracellular DENV particles that exist as mosaic particles can vary substantially depending on various factors, such as the specific DENV strain or the host cell in which the virus was produced (Van Der Schaar et al., 2008; Junjhon et al., 2010; Plevka et al., 2011). We suggest that the maturation state of any live-attenuated strain of DENV used as a dengue vaccine may be critical to its ability to induce protective Ab responses without creating serum conditions that increase the risk of severe dengue disease.

## Data Availability

All datasets generated for this study are included in the manuscript and/or the Supplementary Files.

## Author Contributions

DR developed the stochastic model for antibody binding and carried out the simulations. DR, AW, and SC designed the computational experiments, analyzed the resulting data, and prepared the manuscript.

### Conflict of Interest Statement

The authors declare that the research was conducted in the absence of any commercial or financial relationships that could be construed as a potential conflict of interest.
