# Supplementary material for: Molecular Simulations Reveal the Role of Antibody Fine Specificity and Viral Maturation State on Antibody-Dependent Enhancement of Infection in Dengue Virus
Source: Front Cell Infect Microbiol. 2019 Jun 6;9:200. doi: 10.3389/fcimb.2019.00200 (PMC6593287; doi:10.3389/fcimb.2019.00200)
Supplement: Supplementary file 1 [file Data_Sheet_1.docx]

**Supplemental Materials**

# Molecular simulations reveal role of antibody fine specificity and viral maturation state on antibody-dependent enhancement of infection in dengue virus

Daniel R. Ripoll^1,2^, Anders Wallqvist^2^, and Sidhartha Chaudhury^2^*

1. Henry M. Jackson Foundation for the Advancement of Military Medicine, Rockville, MD
2. Biotechnology HPC Software Applications Institute, Telemedicine and Advanced Technology Research Center, U.S. Army Medical Research and Materiel Command, Fort Detrick, Maryland, USA

# Supplementary Data

**Modeling Ab-virus and Ab-Ab interactions**

## *Ab-virus interactions*

We use the theory of multiple equilibria in proteins to model Ab binding ([1-3](#_ENREF_1)). Abs represent the ligands, and the virus envelope is the macromolecule whose binding sites correspond to the Abs’ epitopes. We model the behavior of a mixture of two types of Abs (indicated as A and B) by adapting a methodology used to study pH titration in proteins ([4](#_ENREF_4)).

A binding state of the viral envelope with N sites is described by a vector$x=(x_{1}^{A}, x_{2}^{A}, \cdots, x_{N_{A}}^{A},x_{1}^{B},x_{2}^{B},\ldots,x_{N_{B}}^{B})$, where $x_{\xi}^{Q}$represents the state of the binding site or epitope ξ, associated with Ab type *Q (Q =* A or B); *N= N_A_ +N_B_* represents the total number of epitopes or binding sites in the envelope; and *N_A_* and *N_B_* correspond to the number of sites of type A and B, respectively. $x_{\xi}^{Q}$ is evaluated as follows:

$x_{\xi}^{Q}= \left\{ \begin{aligned} 1, if an Ab of type Q is bound to the site \\ \\ 0, if the epitope is free \\ \end{aligned} \right.$

The free energy G(***x***) associated with the ***x*** state of the envelope is given by the following expression:

$$G\left( \boldsymbol{x} \right) = \sum_{\xi=1}^{N_{A}} x_{\xi}^{A}(\varepsilon_{\xi}^{A}-\mu_{Ab}^{A})+ \sum_{\xi=1}^{N_{B}} x_{\xi}^{B}(\varepsilon_{\xi}^{B}-\mu_{Ab}^{B}) +$$

$$\frac{1}{2} \sum_{\xi=1}^{N_{A}} \sum_{\eta=1}^{N_{A}} {{W_{\xi\eta} x}_{\xi}^{A} x}_{\eta}^{A}+\frac{1}{2} \sum_{\xi=1}^{N_{B}} \sum_{\eta=1}^{N_{B}} {{W_{\xi\eta} x}_{\xi}^{B} x}_{\eta}^{B}+$$

$$\frac{1}{2} \sum_{\xi=1}^{N_{A}} \sum_{\eta=1}^{N_{B}} {{W_{\xi\eta} x}_{\xi}^{A} x}_{\eta}^{B} (2)$$

where $\varepsilon_{\xi}^{Q}$ represents the intrinsic free energy of association of the Ab to site ξ; $\mu_{Ab}^{Q}$ is the chemical potential of the Ab in solution, and $W_{\xi\eta}$ is the interaction energy between a pair of Abs bound to sites ξ and η.

We consider the epitope site ξ as a collection of pixels or sub sites. When the center of the Ab coincides with any of these sub sites, the Ab is considered bound. The free energy of association of an Ab bound to site ξ is defined by $\varepsilon_{\xi}^{Q}= {\varepsilon͂}_{\xi}^{Q}+ w_{\xi}^{Q}(\Delta r)$, where ${\varepsilon͂}_{\xi}^{Q}$ represents the minimum free energy due to the attachment of Ab type *Q*; and $w_{\xi}^{Q}(\Delta r)$ is the free energy due to formation of H-bonds and other types of favorable interactions between the Ab and residues from the virus at site ξ. $w_{\xi}^{Q}(\Delta r)$ is a function the distance $\Delta r= \left| q_{\xi} \right.-\left. p \right|$ between the Ab position, *p*, and the epitope center $q_{\xi}$ (see Supporting Methods - item *c*). Under this assumption, eq. (2) becomes:

$$G\left( \boldsymbol{x} \right)=\sum_{\xi=1}^{N_{A}} x_{\xi}^{A}({\varepsilon͂}_{\xi}^{A}-\mu_{Ab}^{A})+\sum_{\xi=1}^{N_{A}} x_{\xi}^{A}w_{\xi}^{A}(\Delta r)+$$

$$\sum_{\xi=1}^{N_{B}} x_{\xi}^{B}({\varepsilon͂}_{\xi}^{B}-\mu_{Ab}^{B}) +\sum_{\xi=1}^{N_{B}} x_{\xi}^{B}w_{\xi}^{B}(\Delta r)+$$

$$\frac{1}{2} \sum_{\xi=1}^{N_{A}} \sum_{\eta=1}^{N_{A}} {{W_{\xi\eta}(A,A) x}_{\xi}^{A} x}_{\eta}^{A}+\frac{1}{2} \sum_{\xi=1}^{N_{B}} \sum_{\eta=1}^{N_{B}} {{W_{\xi\eta}(B,B) x}_{\xi}^{B} x}_{\eta}^{B}+$$

$$\frac{1}{2} \sum_{\xi=1}^{N_{A}} \sum_{\eta=1}^{N_{B}} {{W_{\xi\eta}\left( A,B \right)x}_{\xi}^{A} x}_{\eta}^{B} (3)$$

The term${\varepsilon͂}_{\xi}^{Q}-\mu_{Ab}^{Q}$in eq. (3) is related to the Ab-epitope intrinsic dissociation constant,$K_{intr, \xi}^{Q}$, and the concentration, ${[Ab}^{Q}$], of free Ab type *Q*, by the following equation:

$${\varepsilon͂}_{\xi}^{Q}-\mu_{Ab}^{Q}=RT ln10 \left( p{Ab}^{Q}-pK_{intr, \xi}^{Q} \right) (4)$$

Where R is the gas constant; T is the temperature;$p{Ab}^{Q}=- {log}_{10}[{Ab}^{Q}]$; and $pK_{intr, \xi}^{Q}=- {log}_{10}K_{intr, \xi}^{Q}= -\varepsilon_{\xi}^{Q}/ (2.303 RT)$. All epitopes on the virus envelope associated with a given Ab type are considered equivalent, i.e., $K_{intr, \xi}^{Q}=K^{Q}$.

## *Ab-Ab interactions*

We follow the approach introduced by Adamczyk *et al.* ([5](#_ENREF_5), [6](#_ENREF_6)) to account for the steric effects between bound pairs of Abs, ${Ab}^{Q}$ and${Ab}^{Q'}$, and defined the Ab-Ab interaction energy term, $W_{\xi\eta}(Q,Q')$, as:

$W_{\xi\eta}\left( Q,Q^{'} \right)=W_{0}\left( Q,Q^{'} \right) \frac{2}{2+H_{\xi\eta}} e^{-{\kappa a H}_{\xi\eta}} (5)$

Where$H_{\xi\eta}=r_{\xi\eta}-2r_{Fab}$ represents the minimum separation between interacting Abs; $r_{\xi\eta}$ is the distance between centers of a pair of Abs bound at sites ξ and η; $W_{0}\left( Q,Q^{'} \right)$ is the interaction energy at zero separation between Abs type $Q$ and$Q^{'}$; and $\kappa a$ is a parameter that characterizes the "hardness" of the binding Ab;$\kappa a \to\infty$ corresponds to the limiting *hard-sphere* behavior, while finite but decreasing values are associated with Ab particles that become softer. All simulations in this work were carried out assuming a limiting *hard-sphere* behavior, with parameters $W_{0}\left( A,A \right)$ = $W_{0}\left( B,B \right)=W_{0}\left( A,B \right)$ = 200.0 kcal/mol, and *κa* =100Å^-1^.

## Supplementary Figures


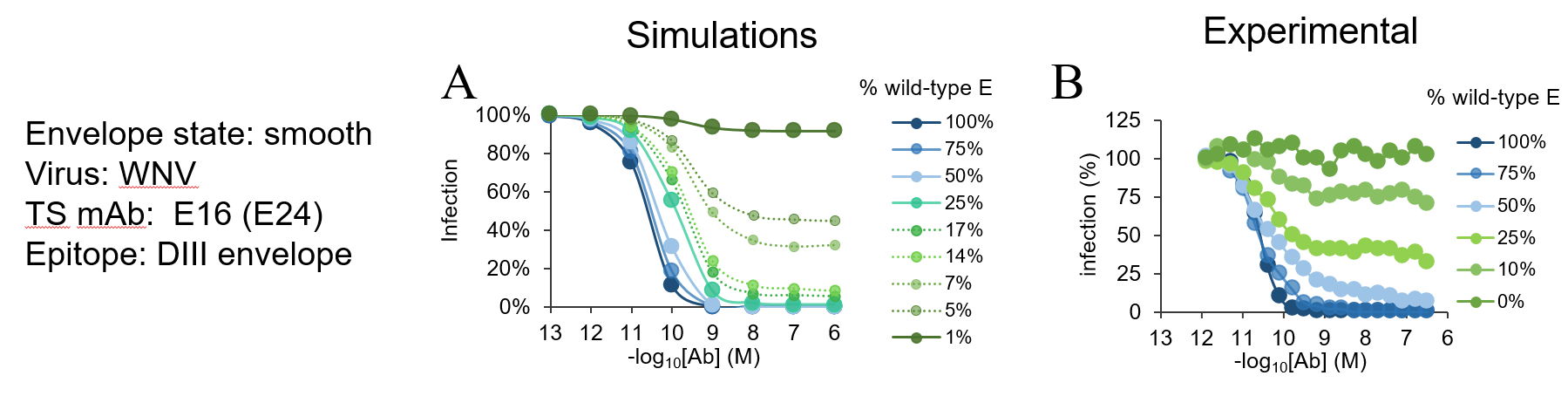


# Supplementary Figure 1. Effects of epitope exposure on WNV Infection. A) Experimentally-observed infection of WNV reporter virus particles (RVPs) as function of the concentration of mAb E24, for varying epitope exposure. Epitope exposure was controlled by incorporating varying proportions of both wild-type E and mutant E proteins that abrogate mAb E24 binding into the RVPs. Data points were extracted from Fig. 5A of Pierson *et al*. ([7](#_ENREF_7)). B) Computed infectivity curves from simulations for virions in the smooth state, assuming virus envelope formed by different ratios of wild-type to mutated E proteins. Epitope information was derived from structural data of the complex WNV – mAb E16 ([8](#_ENREF_8)).

# REFERENCES

1. Edsall, J. T., and J. Wyman. 1958. Biophysical Chemistry. Academic Press, New York, USA.

2. Bisswanger, H. 2008. Enzyme kinetics. Principles and methods. WILEY-VCH Verlag GmbH & Co. KGaA, Weinheim.

3. Steinhardt, J., and J. A. Reynolds. 1969. Multiple Equilibria in Proteins. Academic Press, New York and London.

4. Beroza, P., D. R. Fredkin, M. Y. Okamura, and G. Feher. 1991. Protonation of interacting residues in a protein by a Monte Carlo method: Application to lysozyme and the photosynthetic reaction center of *Rhodobacter sphaeroides*. Proc Natl Acad Sci USA 88:5804-5808.

5. Adamczyk, Z., M. Zembala, B. Siwek, and P. Warszyński. 1990. Structure and ordering in localized adsorption of particles. J. Colloid Interface Sci. 140:123-137.

6. Adamczyk, Z., and P. Belouschek. 1991. Localized adsorption of particles on spherical and cylindrical Interfaces. J. Colloid Interface Sci. 146:123-136.

7. Pierson, T. C., Q. Xu, S. Nelson, T. Oliphant, G. E. Nybakken, D. H. Fremont, and M. S. Diamond. 2007. The stoichiometry of antibody-mediated neutralization and enhancement of West Nile virus infection. Cell Host Microbe 1:135-145.

8. Nybakken, G. E., T. Oliphant, S. Johnson, S. Burke, M. S. Diamond, and D. H. Fremont. 2005. Structural basis of West Nile virus neutralization by a therapeutic antibody. Nature 437:764-769.
